# Combination of Small Molecule Microarray and Confocal Microscopy Techniques for Live Cell Staining Fluorescent Dye Discovery

**DOI:** 10.3390/molecules18089999

**Published:** 2013-08-20

**Authors:** Eszter Molnár, Soujanya Kuntam, Pradeep Kumar Reddy Cingaram, Begüm Peksel, Bhavyashree Suresh, Gabriella Fábián, Liliána Z. Fehér, Attila Bokros, Ágnes Medgyesi, Ferhan Ayaydin, László G. Puskás

**Affiliations:** 1 Laboratory of Functional Genomics, Institute of Genetics, Biological Research Center, Hungarian Academy of Sciences, Temesvári krt. 62, Szeged H-6726, Hungary; 2 Avicor Ltd., Alsó kikötő sor 11, Szeged H-6726, Hungary; 3 Cellular Imaging Laboratory, Biological Research Center, Hungarian Academy of Sciences, Temesvári krt. 62., Szeged H-6726, Hungary; 4 Avidin Ltd., Alsó kikötő sor 11, Szeged H-6726, Hungary

**Keywords:** fluorescent dye discovery, high-throughput screening, small molecule microarray, confocal laser scanning microscopy

## Abstract

Discovering new fluorochromes is significantly advanced by high-throughput screening (HTS) methods. In the present study a combination of small molecule microarray (SMM) prescreening and confocal laser scanning microscopy (CLSM) was developed in order to discover novel cell staining fluorescent dyes. Compounds with high native fluorescence were selected from a 14,585-member library and further tested on living cells under the microscope. Eleven compartment-specific, cell-permeable (or plasma membrane-targeted) fluorochromes were identified. Their cytotoxicity was tested and found that between 1–10 micromolar range, they were non-toxic even during long-term incubations.

## 1. Introduction

Fluorescent labels are widely used to answer questions related to the presence, localization, function, regulation and interactions of specific biomolecules in cells. Their fields of application include immunohisto- and cytochemistry, cell tracking, receptor binding, fluorescence *in situ* hybridization and many others. In addition, labels of this type can play a key role in the validation phase of the protein target identification challenge.

In fluorescence imaging studies, fluorescent proteins (FPs), small molecule dyes (SMDs), chromophoric agents and nanoparticles are commonly used to label cellular compartments, subcellular moieties for both basic and applied research including clinical investigations [1,2].

If a protein marker with characteristic expression for a certain biological pathway is available, it can be visualized by creating a specific, labeled antibody to it or expressing it as an FP-fusion protein. However, both of these techniques have limitations: Antibody labeling usually require the fixation of cells, thus provides no information on the dynamics of the cellular system, while fusion tags might interfere with the function or localization of the protein of interest [3].

SMDs (alias fluorochromes) are substantially smaller in size than FPs. High membrane permeability, less or no toxicity, minimal technical limitations and moderate cost also make SMDs a prominent tool to analyze cellular processes in an unperturbed environment. Although a large number of fluorochromes are known and commercially available by now, there is still a demand for novel molecules marking new targets or having improved spectral characteristics. However, designing molecules for a particular objective is a time-consuming and expensive process requiring high computational power, expertise and often elaborate chemical synthesis [4]. Alternatively, random libraries can be subjected to high-throughput screening (HTS) methods, by which novel dyes with extraordinary characteristics can be identified in a relatively inexpensive and rapid way by simultaneous testing of a large number of compounds, up to hundreds of thousands, in amounts as low as microliters [5]. While these techniques are routinely used in pharmaceutical research, there are only a limited number of applications in basic science for fluorescent probe discovery [6,7,8,9].

Microarrays are powerful HTS tools. Similarly to a DNA or a protein microarray, a small molecule microarray (SMM) is a collection of molecules spotted or synthesized on a solid surface in order to fish out possible binding partners [10,11,12]. This format enables a rapid screen of drug candidates from thousands of small organic compounds that specifically bind to certain proteins marked fluorescently to detect their interaction [11,13,14,15,16,17]. Nevertheless, applicability of the selected compounds under *in vivo* conditions can not be assured in advance.

In the course of the quality control process of SMMs, autofluorescent molecules are to be eliminated or ignored at the subsequent data analysis stage as false positives, since they might interfere with the labeling of the studied protein sample. Thus, compounds showing strong native fluorescence drop out of drug screening, although they still have the potential to be fluorescent probes.

Confocal laser scanning microscopy (CLSM) is one of the most ubiquitous research tools in basic cell biology. Mobility, dynamics, interaction and localization of living cells, organelles and even single molecules can be recorded with high precision due to its high resolution, sensitivity and optical sectioning capability. However, time-consuming manual analysis of images made traditional microscopy a non-scalable method, until the advent of high content microscopy systems opened an accelerated way for screening of multitudinous samples [18]. Still, this technique has some drawbacks like difficulties in automated image analysis and higher cost compared to SMMs.

Therefore we decided to combine the advantages of two approaches for the discovery of new fluorescent dyes, in a chemical library of thousands of diverse compounds. In-house produced SMMs were screened for highly autofluorescent chemicals, which were subsequently investigated on living cells by CLSM. The developed combinational method yielded several new live cell-staining, compartment-specific fluorescent molecules supporting our initial concept.

## 2. Results and Discussion

### 2.1. SMM Prescreening

**Figure 1:**
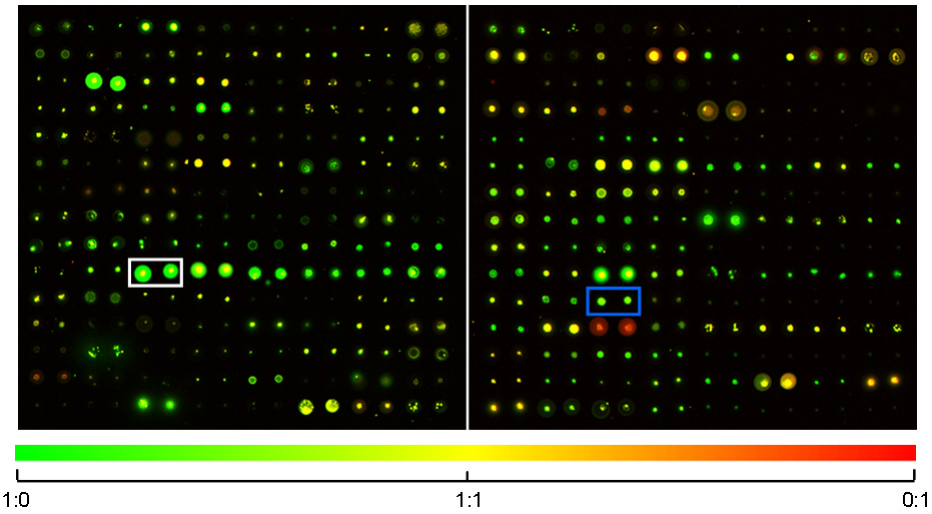
Pseudocolored images of two SMM blocks excited by 532 and 633 nm lasers. Color of the spots derives from the relative emission intensities at 570 and 670 nm (see gradient at the bottom of the Figure). Rectangles show parallel spots of two selected chemicals (white: D10, blue: E5).

14,585 compounds spotted in duplicates or quadruplicates onto chemically activated glass slides were scanned using a dual channel microarray scanner fitted with green (532 nm wavelength) and red (633 nm wavelength) lasers and suitable emission filters (Figure 1). Molecules excitable in wavelengths other than these two are undetectable for the scanner, hence lost for further investigation. This problem can be solved by using a microarray scanner equipped with multiple lasers covering the spectrum from the UV to the far-red region. We restricted our screening approach to dried slides as aqueous analysis would require larger sample volumes to prevent evaporation. Dry analysis not only allowed fast screening of various SMMs, but also proved to be practical from the point of storage convenience for future reference.

The location of spots in the resulting image was defined and statistical parameters of each feature (signal) and background intensities were extracted. The data was filtered for un-flagged (detected) spots, signal to noise ratio (>2), significance (at least 60% of feature pixels with intensities more than two standard deviations above the background) and signal (background corrected median feature intensity >500) in both 532 and 633 nm wavelengths each. Those molecules were selected for microscopic investigation that fulfilled all filtering criteria with two (in case of duplicates) or three (in case of quadruplicates) parallel spots at 532 and/or 633 nm wavelengths (see two of the selected compounds in Figure 1).

### 2.2. *In Vivo* Microscopy Studies

Even though autofluorescent compounds were preselected using 532 and 633 nm lasers, we analyzed them with all four available lasers of the CLSM (405, 488, 543 and 633 nm) as certain fluorochromes may have bimodal excitation or emission peaks. For example, R-phycoerythrin displays an absorption spectrum with distinct peaks at 480 and 565 nm. Moreover, various fluorescent probes change their spectral characteristics due to interaction with organic molecules found in living cells. A well-known example is the nucleic acid intercalating dye acridine orange which has emission peaks at 530 nm or 640 nm based on its interaction with DNA or RNA, respectively. Similarly, intracellular pH, ion concentrations and compartmentalization can also significantly affect the spectral properties of some fluorochromes [19].

278 fluorescent compounds identified via SMMs were first screened for fluorescence intensity, cell permeability, solubility and toxicity under *in vivo* conditions in human HeLa cells using CLSM. Cell impermeable, toxic or low solubility chemicals were eliminated. Some of the dyes had extremely low fluorescence emission when tested in living cells. This may be due to low permeability or interaction with other organic molecules in the culture medium or inside the cells. These chemicals were also disregarded, because the high laser excitation energies they required to obtain detectable fluorescence are not desirable in live cell analyses due to their negative effect on cell metabolism and viability. Following this stringent elimination procedure, we have focused on 11 fluorescent chemicals as novel candidates for live cell imaging (Table 1).

**Figure 2:**
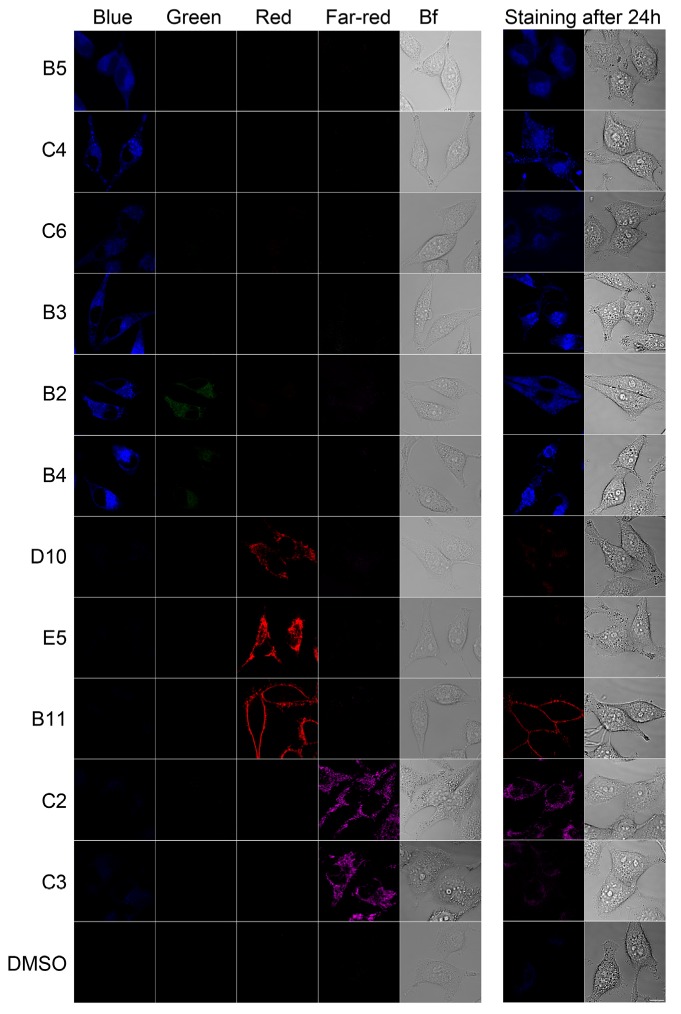
Fluorescence emission and intracellular localization of selected chemicals. Fluorescence emission of the tested chemicals (1 h at 37 °C, 10 µM each, except C2 which was 1 µM) were captured in four channels (blue, green, red and far-red) using 405, 488, 543 and 633 nm laser excitation. Fluorescence emissions were also captured after 1 day of incubation at 37 °C (staining after 24 h). Only the image of the brightest fluorescence color paired with the corresponding bright field image was shown for “Staining after 24h” images. Up to three times wider confocal aperture (pinhole) was used for taking “Staining after 24h” images. Control cells were treated with DMSO. Bf: Bright field imaging. Scale bar 10 µm.

Four channel emission images of the 11 compounds are shown in Figure 2. We have found five chemicals localized to lipid droplets (LDs), three to mitochondria, one to plasma membrane, one to perinuclear/cytoplasmic region and one to mitochondria/nucleolus (Table 1 and Figure 2). Laser excitation and emission detection thresholds for microscopy imaging were set by using dimethyl sulfoxide (DMSO)-treated cells as reference (Figure 2). All of the chemicals were labeling the target compartment within 1 h of incubation at 37 °C when used at 10 µM. Chemical C2 was very bright and detectable, even at a concentration as low as 0.25 µM, however, it was not well tolerated by the cells at 10 µM, therefore its use was limited to a maximum of 1 µM. We have also tested stability and labeling persistency of these chemicals. Apart from chemicals E5, D10 and C3, they were still fluorescent even after one day of incubation at 37 °C (Figure 2).

**Table 1:** Chemical structures and labeling characteristics of the selected molecules.

ID	Structure, Localization, Color	ID	Structure, Localization, Color
B5	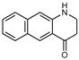 Perinuclear/cytoplasm (blue)	D10	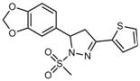 Mitochondria (red)
C4	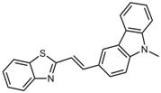 Lipid droplets (blue)	E5	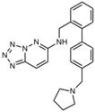 Mitochondria (red)
C6	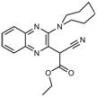 Lipid droplets (blue)	B11	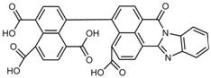 Plasma membrane (red)
B3	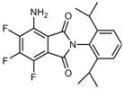 Lipid droplets (blue)	C2	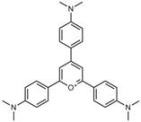 Mitochondria/nucleolus convertible(far-red)
B2	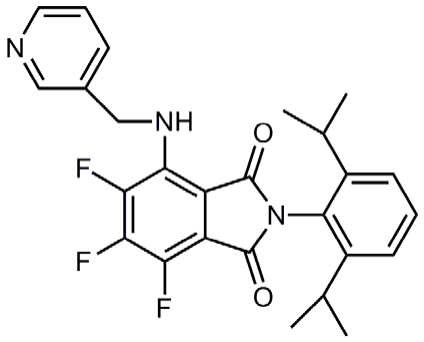 Lipid droplets (blue-green)	C3	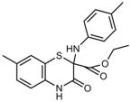 Mitochondria (far-red)
B4	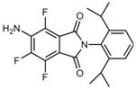 Lipid droplets (blue-green)	DMSO	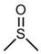 Solvent used as control (colorless)

**Figure 3:**
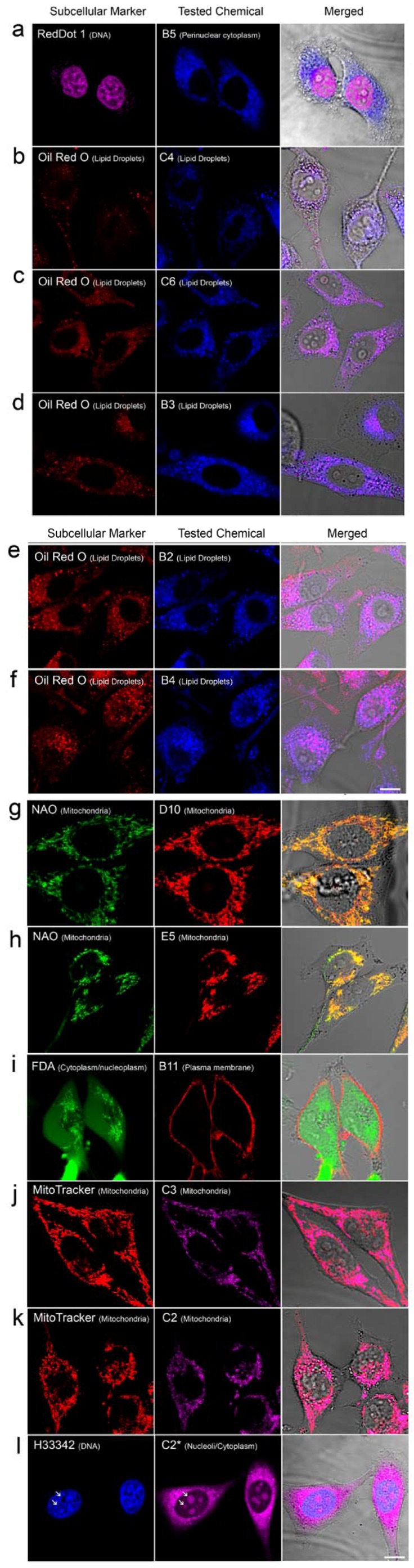
Colocalization of chemicals with fluorescent markers. Localization of the chemicals and the fluorescent markers are indicated on Figures. (**b**–**f**) Formaldehyde fixed cells were used for colocalization with Oil Red O. Live cell colocalizations were shown in (**a** and **g**–**l**). (**l**) Laser excitation-induced relocalization of chemical C2 to nucleolus/cytoplasm. NAO: Nonyl acridine orange; FDA: Fluorescein diacetate; H33342: Hoechst 33342; C2*: Photoconverted form of chemical C2. Arrows indicate two nucleoli. Scale bar 10 µm.

Intracellular localization of the autofluorescent compounds was determined by colocalization with known fluorescent markers (Figure 3a–l). Figure 3a shows colocalization of a DNA specific nuclear marker dye RedDot 1 with chemical B5 which is localized to the perinuclear region. Figure 3b–f show Oil Red O staining of LDs colocalizing with chemicals C4, C6, B2, B3 and B4 on fixed HeLa cells. Chemicals B2, B3 and B4 are derived from our in-house non-randomly synthesized collection along with some other trifluoroaminophthalimide derivatives, which were further developed as potential anticancer agents [20]. Therefore molecules B2–B4 (Table 1) have the same basic structure in contrast with the other eight fluorochromes from commercially available libraries. Unlike Oil Red O stain, all five of the LD-localized novel fluorescent probes were cell-permeable dyes without the necessity of cell fixation, hence allowing live analysis of LD localization, mobility and dynamics. Figure 3g–k show five other fluorescent live cell markers discovered in this study. Chemicals D10, E5, C2 and C3 were all localized to mitochondria. Co-staining of red-colored D10 and E5 with nonyl acridine orange (NAO, green) (Figure 3g,h) and far-red colored chemicals C2, C3 with MitoTracker Orange (red) (Figure 3j,k) resulted in complete colocalization with these mitochondria localized markers. Chemical B11 localizes preferentially to plasma membrane (Figure 3i). Cell borders labeled with this chemical were still visible even after one day of incubation in culture without significant loss of cell viability (Figure 2), therefore this dye can also be used in long-term live cell labeling and tracking experiments. The localization of chemical B11 was confirmed by co-staining with the whole cell-labeling fluorescein diacetate (FDA) (Figure 3i).

**Figure 4:**
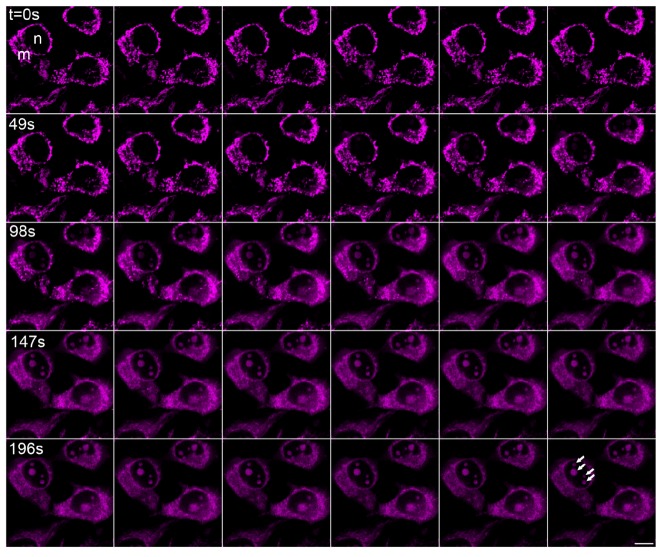
Laser scanning-induced relocalization of chemical C2 from mitochondria to cytoplasm and nucleoli. C2-treated (1 µM) HeLa cells were repeatedly scanned with 633 nm HeNe laser at 50% intensity. Each frame is 7 seconds apart. Elapsed time is shown for every 7^th^ frame. Mitochondria (m) and nucleus (n) of a cell are shown at the first frame. The four nucleoli of the same cell are indicated with arrows at the last frame. Bar 10 µm.

**Figure 5:**
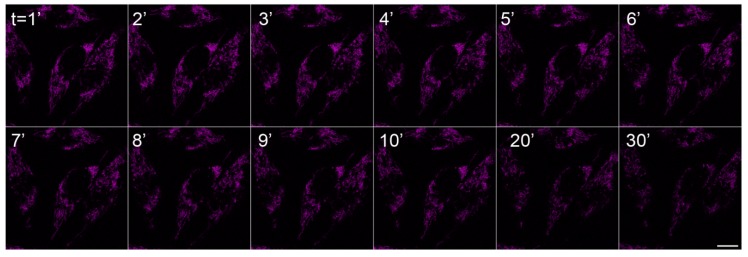
Stability of chemical C2 using low laser intensity settings. 633 nm laser was used at 4% intensity to capture images at indicated time points. Bar 10 µm.

**Figure 6:**
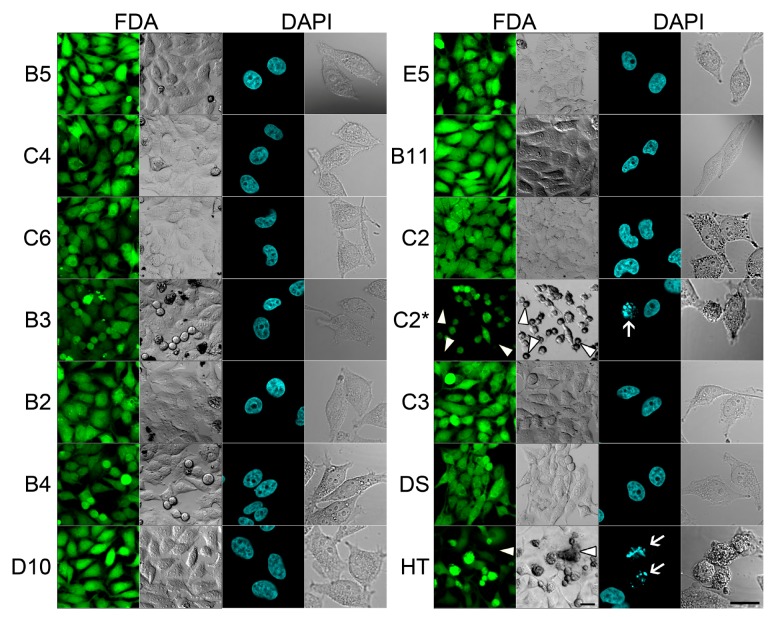
Viability and nuclear morphology analyses of 24 h-treated HeLa cells. FDA (green) labeling of live cells and DAPI (blue) labeling of fixed cells and their corresponding bright field images (black and white transmitted light images) are shown. Arrowheads in C2* and HT samples indicate FDA-negative dead cells and clusters. Arrows show fragmented, apoptotic-like nuclei in C2* and HT samples. C2*: Photoconverted C2; DS: Dimethyl sulfoxide; HT: 4-hydroxytamoxifen. Scale bars of 20 µm are shown in HT samples for both FDA and DAPI image sets.

During microscopy analyses, we have discovered an unusual spectral behavior of the chemical C2. Repetitive scanning with 633 nm at 50% intensity setting caused gradual relocalization of dye signal from mitochondria to cytoplasm/nucleolus as shown in Figure 4. Using a lower laser intensity setting (4%) for the 633 nm laser did not cause this conversion indicating that the mitochondria-localized C2 dye was not changing its properties by itself and that certain threshold amount of laser energy is necessary to cause relocalization (Figure 5). Photoconversion was also inducible by illumination with the rhodamine filter set of the fluorescence microscope (510–550 nm green excitation). To confirm the nucleolar localization of the photoconverted C2 dye (C2*), we have used co-staining on live cells 1 h after photoconversion with a vital DNA dye Hoechst 33342 which displays a negative staining pattern at the RNA-rich nucleolar regions (arrows in Figure 3l). We predict that, like photoconvertible and photosensitizing fluorescent proteins [19], this chemical may also undergo laser-induced structural changes which may cause its relocalization. Upon light stimulation, the pyrylium core of C2 is known to behave as an electron acceptor [21] which is capable of oxidizing surrounding donor molecules within the cell (photooxidation). Previously it has been reported that photosensitizing fluorescent probes may cause dye relocalization due to cell death [22]. Light-induced relocalization of chemical C2 may also be due to similar photosensitization and toxicity. Reduced viability of chemical C2*-treated cells after 1 day of incubation (Figure 6) also supports this possibility. However in the absence of laser excitation for the photoconversion of C2 molecule, it stayed stable in mitochondria for longer period of time (Figure 2). Successive laser scanning using reduced laser intensity (633 nm laser at 4%) did not cause photoconversion, either (Figure 5).

We have also tested the effect of these chemicals on long-term viability of cultured cells. As a positive control, 4-hydroxytamoxifen (HT), a known apoptosis inducer was used at a concentration of 20 µM. HeLa cells were treated with chemicals at a concentration of 10 µM (1 µM for chemicals C2 and C2*) during a 24 h period. Viability was assessed using FDA staining. Due to esterase activity, living cells will actively convert the non-fluorescent FDA into the bright green fluorescent compound fluorescein [19]. Figure 6 shows that 24 h treatment did not significantly affect viability, except C2* and HT, as compared to DMSO-treated cells. Hence these dyes are also suitable for long-term labeling and tracking experiments. Although C2* did not induce any visible morphological changes during 1 h postconversion (Figure 3l), 24 h treatment significantly caused cell round-up with reduced viability (arrowheads on Figure 6 C2* indicate dead cells). Therefore, we concluded that the application of C2* is limited to short term (such as 1 h) colocalization analyses.

As an alternative approach, the chemicals were also tested for apoptotic nuclei formation. Apoptotic cells display distinct nuclear morphological changes such as nuclear blebbing and degradation of nuclear envelope [23]. Cells treated with HT at 20 µM and with compounds at 10 µM (1 µM for C2 and C2*) for 24 h were fixed and stained with DAPI to screen nuclear morphology changes. As shown in Figure 6, none of the chemicals displayed nuclear abnormalities except C2* and HT for which blebs and micronuclei were frequently observed (Figure 6, DAPI image of C2* and HT).

## 3. Experimental Section

### 3.1. Materials

The 14,585-member small molecule collection consisted of two commercially available libraries: 8,800 compounds from Nanosyn (Santa Clara, CA, USA) and 5,120 compounds from Enamine (Kiev, Ukraine), and a 665-member library of Avidin Ltd. (Szeged, Hungary). Hoechst 33342 and MitoTracker Orange were purchased from Invitrogen (Grand Island, NY, USA), Dulbecco’s modified Eagle's medium (DMEM), antibiotics and trypsin from Lonza (Basel, Switzerland), RedDot 1 from Biotium (Hayward, CA, USA), all others were from Sigma-Aldrich (St. Louis, MO, USA).

### 3.2. Small Molecule Microarray (SMM)

#### 3.2.1. Preparation

Compounds (20 µL each in 384-well polypropylene plates) were prepared as DMSO solutions at a concentration of 5 mM. For printing chemically activated glass slides (AviChemix from Avicor Ltd., Szeged, Hungary) were used, which contain a treelike, branched dendrimer structure with reactive functional groups at the terminal position [10,12]. Printing was performed by using a MicroGrid II (BioRobotics, Cambridge, UK) mechanical microarray microspotter with split pins at room temperature under 50% humidity. Chemicals were spotted in duplicates or quadruplicates, and the diameter of each spot was approximately 150 µm.

#### 3.2.2. Signal Detection and Data Analysis

After spotting the microarrays were scanned using an Agilent G2505B Microarray Scanner (lasers: 532 nm SH-YAG and 633 nm HeNe) with 10 µm resolution and maximum laser power. Microarray images were analyzed by the Feature Extraction Software (Agilent, Waldbronn, Germany) and GenePix Pro 6.0 (Axon Instruments, Foster City, CA, USA).

### 3.3. Cell Culturing

Human HeLa cervical carcinoma cells were cultured in DMEM supplemented with 10% fetal bovine serum, 2 mM glutamine and an antibiotic mixture (100 units/mL penicillin and 100 µg/mL streptomycin) at 37 °C under 5% CO_2_ and 85% humidity.

### 3.4. Cell treatment

#### 3.4.1. Treatment with Selected Compounds

One day after plating, cell cultures were treated with the chemicals at 1 and 10 µM for 1–2 h (short incubation) or one day (long-term incubation) on a 96-well glass bottom microplate (Greiner Bio-One, Kremsmünster, Austria) or LabTek 8-well coverslip-bottom chambers (Nalge Nunc International, Rochester, NY, USA). Photoconversion of chemical C2 was achieved either via successive scanning with 633 nm HeNe laser at 50% laser intensity setting or using the rhodamine filter set excitation (510–550 nm) for 10 s using maximum intensity of HBO 103W/2 (Osram, Munich, Germany) mercury short arc burner. Stability of chemical C2 at low laser intensity was recorded using successive scanning with 633 nm laser at 4% laser intensity setting (Figure 5). Control cultures were treated with 1% DMSO.

#### 3.4.2. Co-Labeling

DNA intercalating dye Hoechst 33342 was used at 1 µM to stain live cell nuclei. MitoTracker Orange (0.2 µM) and nonyl acridine orange (0.5 µM) were used to label mitochondria. Fluorescein diacetate (FDA) was used at 0.1 µM to highlight the cells. RedDot 1 is supplied as 200 × concentrated solution and used by diluting 200 × in culture medium as suggested by the manufacturer. Fixed cells were used for LD colocalization and apoptotic nuclei analysis: Cell fixation was performed using 4% paraformaldehyde in phosphate buffered saline (PBS) for 7 min at ambient temperature. Fixer was removed with 3 × 5 min PBS washes. For LD colocalization Oil Red O was prepared as 1% (w/v) in isopropanol. Cells were first incubated with the tested fluorochromes for 10 min then Oil Red O was added at a final concentration of 0.01% (w/v) at the center of the chamber. Regions surrounding the isopropanol-exposed central part were analyzed for colocalization. For apoptotic analysis HeLa cells were incubated for 24 h with the chemicals at 10 µM (1 µM for C2 and C2*). Following PBS washes, cells were fixed and stained with 0.1 µg/mL DAPI for 10 min.

### 3.5. Confocal Laser Scanning Microscopy (CLSM)

CLSM was performed using Olympus Fluoview FV1000 confocal laser scanning microscope (Olympus Life Science Europa GmbH, Hamburg, Germany). Microscope configuration was as follows: Objective lenses: UPLSAPO 20 × (dry, NA:0.75), UPLFLN 40 × (oil, NA:1.3) and UPLSAPO 60 × (oil, NA:1.35); sampling speed: 4 µs/pixel; line averaging: 2 ×; scanning mode: Sequential unidirectional; excitation: 405 nm (Hoechst 33342, DAPI and blue fluorescence detection of the novel chemicals) 488 nm (NAO, FDA and green fluorescence detection), 543 nm (Oil Red O, MitoTracker Orange and red fluorescence detection) and 633 nm (RedDot 1 and far-red fluorescence detection); maximum laser transmissivity values: 2% (405 nm), 5% (488 nm), 50% (543 nm) and 50% (633 nm); emission filters: Blue: 425–475 nm, Green: 500–530 nm; Red: 555–625 nm; Far-red: λ > 650 nm. Before imaging one-day-treated cells, detector pinhole diameter (1 airy unit) was increased two to three times to capture faint dye fluorescence emissions. In Figure 6, blue-colored dyes which had reduced intensity due to 24 h incubation in culture medium and formaldehyde fixation, was not detectable with the microscope settings used for bright DAPI dye detection. Images were pseudocolored using Olympus Fluoview software (version 2.0 c).

## 4. Conclusions

A dual-approach strategy involving both SMM and CLSM was applied in order to identify new cell staining fluorochromes. A 14,585-member chemical library deposited in a microarray format was screened for autofluorescent compounds. *In vivo* microscopy analyses of the selected molecules resulted in the discovery of 11 unique, nontoxic and cell-permeable (or plasma membrane-targeted) fluorescent probes. Further chemical modifications and detailed characterization of the labeling targets of these chemicals present an exciting opportunity to discover a number of novel fluorochromes with enhanced chemical and spectral properties for use in live cell analyses.
